# Echinochrome A Promotes Ex Vivo Expansion of Peripheral Blood-Derived CD34^+^ Cells, Potentially through Downregulation of ROS Production and Activation of the Src-Lyn-p110δ Pathway

**DOI:** 10.3390/md17090526

**Published:** 2019-09-09

**Authors:** Ga-Bin Park, Min-Jung Kim, Elena A. Vasileva, Natalia P. Mishchenko, Sergey A. Fedoreyev, Valentin A. Stonik, Jin Han, Ho Sup Lee, Daejin Kim, Jee-Yeong Jeong

**Affiliations:** 1 Department of Biochemistry, Cancer Research Institute, Kosin University College of Medicine, Busan 49267, Korea (G.B.P.) (M.J.K.); 2 G.B. Elyakov Pacific Institute of Bioorganic Chemistry, Far-Eastern Branch of the Russian Academy of Science, Vladivostok 690022, Russia (E.A.V.) (N.P.M.) (S.A.F.) (V.A.S.); 3 National Research Laboratory for Mitochondrial Signaling, Department of Physiology, Cardiovascular and Metabolic Disease Center, Inje University College of Medicine, Busan 47392, Korea; 4 Department of Internal Medicine, Kosin University College of Medicine, Busan 49267, Korea; 5 Department of Anatomy, Inje University College of Medicine, Busan 47392, Korea

**Keywords:** hematopoietic stem and progenitor cells, CD34^+^ cells, ex vivo expansion, Lyn, Src, p110δ, ROS

## Abstract

Intracellular reactive oxygen species (ROS) play an important role in the proliferation and differentiation of hematopoietic stem and progenitor cells (HSPCs). HSPCs are difficult to be expanded ex vivo while maintaining their stemness when they are exposed to oxidative damage after being released from the bone marrow. There have been efforts to overcome this limitation by using various cytokine cocktails and antioxidants. In this study, we investigated the effects of echinochrome A (Ech A)-a well-established and non-toxic antioxidant-on the ex vivo expansion of HSPCs by analyzing a CD34^+^ cell population and their biological functions. We observed that Ech A-induced suppression of ROS generation and p38-MAPK/JNK phosphorylation causes increased expansion of CD34^+^ cells. Moreover, p38-MAPK/JNK inhibitors SB203580 and SP600125 promoted ex vivo expansion of CD34^+^ cells. We also demonstrated that the activation of Lyn kinase and p110δ is a novel mechanism for Ech A to enhance ex vivo expansion of CD34^+^ cells. Ech A upregulated phospho-Src, phospho-Lyn, and p110δ expression. Furthermore, the Ech A-induced ex vivo expansion of CD34^+^ cells was inhibited by pretreatment with the Src family inhibitor PP1 and p110δ inhibitor CAL-101; PP1 blocked p110δ upregulation and PI3K/Akt activation, whereas CAL-101 and PI3K/Akt pathway inhibitor LY294002 did not block Src/Lyn activation. These results suggest that Ech A initially induces Src/Lyn activation, upregulates p110δ expression, and finally activates the PI3K/Akt pathway. CD34^+^ cells expanded in the presence of Ech A produced equal or more hematopoietic colony-forming cells than unexpanded CD34^+^ cells. In conclusion, Ech A promotes the ex vivo expansion of CD34^+^ cells through Src/Lyn-mediated p110δ expression, suppression of ROS generation, and p38-MAPK/JNK activation. Hence, Ech A is a potential candidate modality for the ex vivo, and possibly in vivo, expansion of CD34^+^ cells.

## 1. Introduction

Hematopoietic stem and progenitor cell (HSPC) transplantation is widely used for the treatment of various hereditary diseases and blood-related malignancies, such as leukemia, and to promote hematologic recovery following anticancer therapy [1]. HSPCs can be harvested from bone marrow, umbilical cord blood (UCB), or mobilized peripheral blood (PB) using granulocyte colony-stimulating factor (G-CSF) administration for autologous and allogeneic transplantation [2]. Currently, PB-HSPC transplantation accounts for more than 60% of total HSPC transplantation worldwide, mainly due to the less invasive collection procedures [3]. However, the insufficient number of HSPCs, even after multiple days of collection, is a limiting factor for their clinical application of transplantation [4]. Several researchers are exploring ex vivo expansion of HSPCs to overcome this limitation; however, ex vivo expansion remains a difficult challenge for HSPC-based therapies.

Reactive oxygen species (ROS) are generated in the mitochondria; they regulate proliferation, differentiation, motility, and quiescence in many cell types, including HSPCs [5,6]. A previous study using a mouse model showed that increased levels of ROS can promote the differentiation of stem cells [7]. Quiescent HSPCs reside in a hypoxic niche in the bone marrow microenvironment that protects them from oxidative stress caused by excessive ROS production, mitochondrial dysfunction, or a combination of both [7,8]. HSPCs can proliferate and differentiate in the oxygen-rich vascular niche, resulting in increased intracellular ROS levels. ROS can regulate HSPC activity and various levels of ROS accumulation may affect the fate of HSPCs. High levels of ROS can trigger HSPC dysfunction, aging, and DNA damage. On the contrary, moderate levels of ROS are necessary for the proliferation, mobilization, and differentiation of HSPCs, and low-ROS cells have been shown to retain long-term self-renewal ability [7,9,10,11]. Culture media with appropriate cytokines are usually required for ex vivo cultures of HSPCs. However, it is still important to regulate the levels of ROS, as cytokine treatment itself triggers intracellular ROS generation [12]. Antioxidants can effectively remove excess ROS and maintain the redox balance of cells [13,14]. A recent study showed that N-acetyl cysteine (NAC) can reduce ROS levels to enhance ex vivo expansion of HSPCs [15].

Src family kinases (SFKs), including Lyn, Fyn, Fgr, Yes, Lck, Hck, Blk, and Trk, are well known for their contribution to malignant transformation and oncogenesis, and control downstream targets to regulate cell proliferation, differentiation, adhesion, migration, and the cell cycle [16]. Because of their role in cancer development and progression, SFKs have become critical targets for cancer therapy [17]. In the immune system, the most well-known function of SFKS is their role in integrin signaling [16]. Fyn and Lck kinases are also found in T cells and natural killer (NK) cells [18]. Lyn is a non-receptor tyrosine kinase that is predominantly found in the hematopoietic cells of myeloid and B lymphocyte lineages [19]. Lyn was originally identified as a hematopoietic-specific kinase; it is expressed in multiple tissues and is involved in the signaling of the B-cell receptor [20], GM-CSF receptor [21], erythropoietin (EPO) receptor [22], and c-kit [23]. Lyn phosphorylates several signaling molecules, including PI3K, FAK [24], ras-GAP, and Stat5 [25]. Lyn also plays an important role in acute myeloid leukemia (AML) cell proliferation [26], and the silencing of Lyn in imatinib-resistant chronic myelogenous leukemia (CML) cells can induce apoptosis [27]. Lyn activation induces p110 expression, whereas Lyn inhibition decreased migration in ovarian cancer cells exposed to cigarette smoke [28]. Colon cancer cells use Lyn for activation of the anti-apoptotic PI3K p110/Akt pathway and the induction of epithelial-mesenchymal transition (EMT) [29]. Therefore, several pieces of evidence show an important association of Lyn in both leukemia and solid tumor development. However, the exact roles and Lyn/Src activation in the relationship with ROS during the ex vivo expansion of HSPCs are still unclear.

Echinochrome A (Ech A) is a dark red pigment that is isolated from eggs, spines, and larvae of sea urchins [30]. Ech A is known to possess antioxidant, antiviral, antialgal, and antimicrobial activities [31,32]. Importantly, Ech A was shown to exhibit diverse intracellular antioxidant mechanisms, including the elimination of free radicals [33], inhibition of pulmonary fibrosis [34], and chelation of metal ions [35]. In this study, we demonstrate that Ech A is an effective agent to promote the ex vivo expansion of G-CSF-mobilized PB-derived CD34^+^ HSPCs through Src/Lyn-mediated p110δ upregulation, the suppression of ROS generation, and p38-MAPK/JNK activation.

## 2. Results

### 2.1. Ech A Suppresses ROS Production and Promotes Expansion of PBMC-Derived CD34^+^ Cells

**Figure 1:**
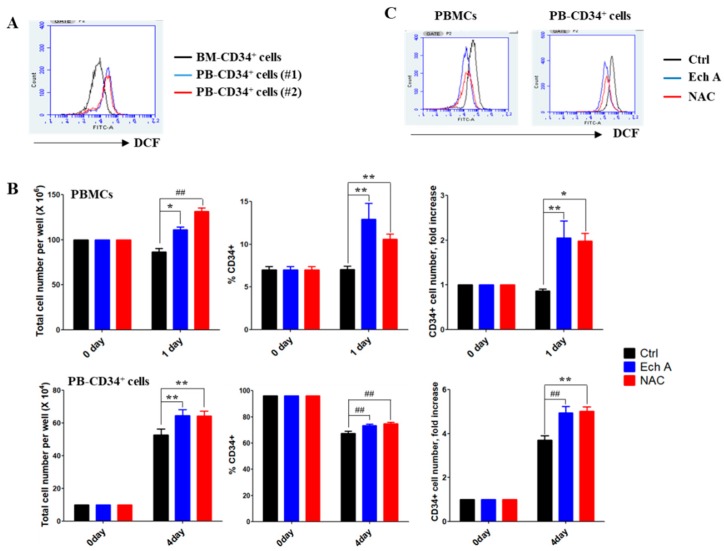
Ech A increases PB-CD34^+^ cell expansion by inhibiting reactive oxygen species’ (ROS) generation. (**A**) Cells were treated with 10 μM 2′7′-dichlorodihydro-fluorescein diacetate for 30 min. The values in the 2′7′-dichlorofluorescein histograms indicate MFI (mean fluorescence intensity). (**B**,**C**) Cells were treated with 10 μM Ech A for 1 day (PB mononuclear cells, PBMCs) or 4 days (PB-CD34^+^ cells). For NAC treatment, cells were treated with 5 mM NAC for 4 h, washed, suspended in complete medium, and incubated for an additional 1 day (PBMCs) or 4 days (PB-CD34^+^ cells). (**B**) Total cell number was measured using the ADAM-MC automated mammalian cell counter (NanoEntech, Seoul, Korea). For flow cytometric immunophenotypic analysis, cells were stained with CD34-PE, CD38-FITC, CD45-APC, and 7-AAD. Each value was expressed as the mean ± SEM of three independent experiments. (**C**) Cells were treated with 10 μM 2′7′-dichlorodihydro-fluorescein diacetate for 30 min and ROS levels were subsequently measured usingthe flow cytometer.

HSPCs reside in a hypoxic niche in the bone marrow, suggesting that HSPCs need to adopt unique metabolic properties, including intracellular ROS levels. We found that PB-derived CD34^+^ cells (PB-CD34^+^ cells) exhibit higher ROS levels than BM-derived CD34^+^ cells (Figure 1A). We then examined whether Ech A can modulate ROS levels to cause an ex vivo expansion of PB-CD34^+^ cells. To determine the optimal concentration of Ech A, G-CSF-mobilized PB mononuclear cells (PBMCs) were treated with different concentrations of Ech A (0, 1, 10, 20, 50, and 100 μM) for 24 h, and the CD34^+^ cell number was analyzed by flow cytometry. Cells treated with 10 μM Ech A for 24 h showed approximately two-fold higher CD34^+^ cell number than those in the control group (Figure S1A); therefore, we chose that condition for subsequent experiments. The effect of Ech A on the ex vivo expansion of PBMCs containing CD34^+^ or purified PB-CD34^+^ was investigated after 1 day or 4 days of culture, respectively (Figure 1B). The toxic reagent N-acetyl cysteine (NAC)—a well-known potent antioxidant—was used as the positive control. Immunophenotypic analysis showed a significantly higher percentage of CD34^+^ cells and the CD34+ cell number in Ech A-treated group (PBMCs, 12.91% ± 3.22%, 2.05 ± 0.66-fold; PB-CD34^+^ cells, 73.37% ± 1.11%, 4.95 ± 0.28-fold) than that in the control group (PBMCs, 7.07 ± 0.66%, 0.87 ± 0.08-fold; PB-CD34^+^ cells, 67.27 ± 1.79%, 3.71 ± 0.18-fold). As shown in Figure 1C, Ech A dramatically suppressed intracellular ROS production in PBMCs and PB-CD34^+^ cells. Additionally, H_2_O_2_ treatment showed dramatic suppression of PB-CD34^+^ cell expansion that was recovered by Ech A treatment (Figure S2). These results suggest that Ech A promotes the ex vivo expansion of PBMC-derived CD34^+^ cells by suppressing ROS levels.

### 2.2. Ech A Inhibits the Activation of p38-MAPK and JNK in PB-CD34^+^ Cells

**Figure 2:**
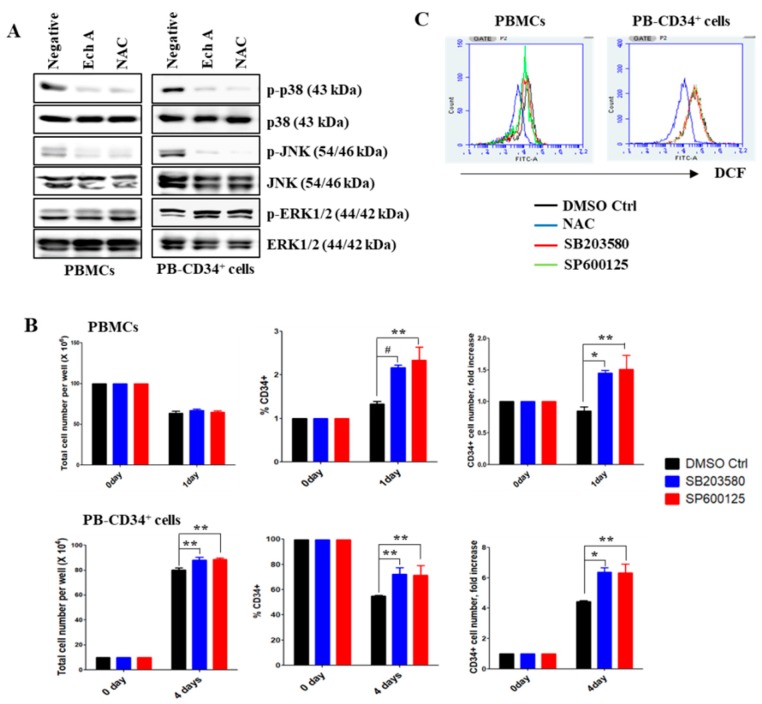
Ech A enhances PB-CD34^+^ cell expansion by suppressing the activation of p38-MAPK and JNK. Cells were treated with 10 μM Ech A for 1 day (PBMCs) or 4 days (PB-CD34^+^ cells). For NAC treatment, cells were treated with 5 mM NAC for 4 h, washed, suspended in complete medium, and incubated for an additional 1 day (PBMCs) or 4 days (PB-CD34^+^ cells). (**A**) Total protein was subjected to western blot analysis with the indicated antibodies. β-actin served as the loading control. (B, C) Cells were pretreated with SB203580 (10 μM) or SP600125 (10 μM) for 4 hr. (**B**) Total cell number was measured using the ADAM-MC automated mammalian cell counter. For flow cytometric immunophenotypic analysis, cells were stained with CD34-PE, CD38-FITC, CD45-APC, and 7-AAD. Each value was expressed as the mean ± SEM of three independent experiments. (**C**) Cells were treated with 10 μM 2′7′-dichlorodihydro-fluorescein diacetate for 30 min. The values in the 2′7′-dichlorofluorescein histograms indicate MFI.

After 1 or 4 days of culture, the levels of phosphorylated p38-MAPK and JNK were significantly increased in the expanded cells (Figure 2A; negative control). However, the phosphorylation of p38-MAPK and JNK was dramatically suppressed upon the treatment of Ech A or NAC (Figure 2A). On the other hand, ERK1/2 phosphorylation remained unchanged in the cells. To examine whether p38-MAPK/JNK activation inhibits the ex vivo expansion of human PB-CD34^+^ cells, cells were cultured in the presence of vehicle (0.1% DMSO), SB203580 (SB; 5 μM), or SP600125 (SP; 5 μM) for 1 or 4 days. The expanded cells were harvested for cell counts, and the analysis of CD34 expression by flow cytometry. As illustrated in Figure 2B, the total number of PBMCs was comparable irrespective of whether cells were incubated with or without SB or SP. Cell cultures with SB or SP showed a considerable increase in the number of CD34^+^ cells compared to the DMSO control group. In particular, the number of CD34^+^ cells was approximately 1.5-fold higher in the presence of SB (PBMCs, 2.17% ± 0.06%, 1.45 ± 0.05-fold; PB-CD34^+^ cells, 72.27% ± 5.01%, 6.38 ± 0.27-fold) or SP (PBMCs, 2.33% ± 0.31%, 1.51 ± 0.22-fold; PB-CD34^+^ cells, 71.27% ± 7.83%, 6.34 ± 0.61-fold) than that in the DMSO control group (PBMCs, 1.33% ± 0.06%, 0.85 ± 0.07-fold; PB-CD34^+^ cells, 55.00% ± 0.56%, 4.43 ± 0.05-fold). However, ROS levels in PB-CD34^+^ cells were unaffected by SB or SP (Figure 2C). These results suggest that p38-MAPK/JNK inhibition increases the ex vivo expansion of CD34^+^ cells, even at high ROS levels.

### 2.3. Ech A Regulates PB-CD34^+^ Cell Expansion via PI3K/Akt Pathway

**Figure 3:**
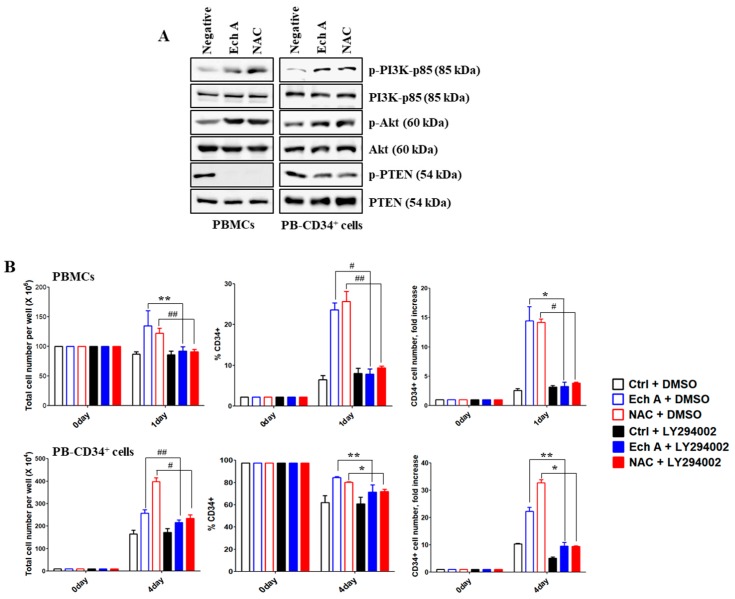
Echinochrome A (Ech A) upregulates PB-CD34^+^ cell expansion by activating PI3K/Akt pathway. Cells were treated with 10 μM Ech A for 1 day (PBMCs) or 4 days (PB-CD34^+^ cells). For NAC treatment, cells were treated with 5 mM NAC for 4 h, washed, suspended in complete medium, and incubated for an additional 1 day (PBMCs) or 4 days (PB-CD34^+^ cells). (**A**) Total cell lysates for each condition were immunoblotted with the indicated antibodies. (**B**) Cell were pretreated with LY294002 (10 μM) for 4 h. Total cell number was determined using the ADAM-MC automated mammalian cell counter. For flow cytometric immunophenotypic analysis, cells were stained with CD34-PE, CD38-FITC, CD45-APC, and 7-AAD. Each value was expressed as the mean ± SEM of three independent experiments.

We further assessed the possible molecular mechanisms underlying Ech A-mediated ex vivo expansion of PB-CD34^+^ cells. Our results showed that phosphorylation of PI3K-p85 and Akt was enhanced to a greater extent in Ech A- or NAC-treated cells than that in untreated cells (Figure 3A); however, phospho-PTEN level were reduced. To verify the requirement of PI3K/Akt signaling activation for PB-CD34^+^ cell expansion, we used the PI3K/Akt inhibitor LY294002. As shown in Figure 3B, pretreatment of the Ech A or NAC treatment group with LY294002 (PBMCs, 7.83 ± 1.31%, 3.29 ± 0.73-fold; PB-CD34^+^ cells, 71.43 ± 6.45%, 9.55 ± 1.41-fold) substantially reduced the percentage of CD34^+^ cells as well as CD34^+^ cell number compared to Ech A or NAC single treatment group (PBMCs, 23.63 ± 1.7%, 14.43 ± 2.42-fold; PB-CD34^+^ cells, 84.43 ± 0.93%, 22.33 ± 1.49-fold).

### 2.4. The PI3K p110δ Isoform Is Required for Ech A-Induced CD34^+^ Cell Expansion

**Figure 4:**
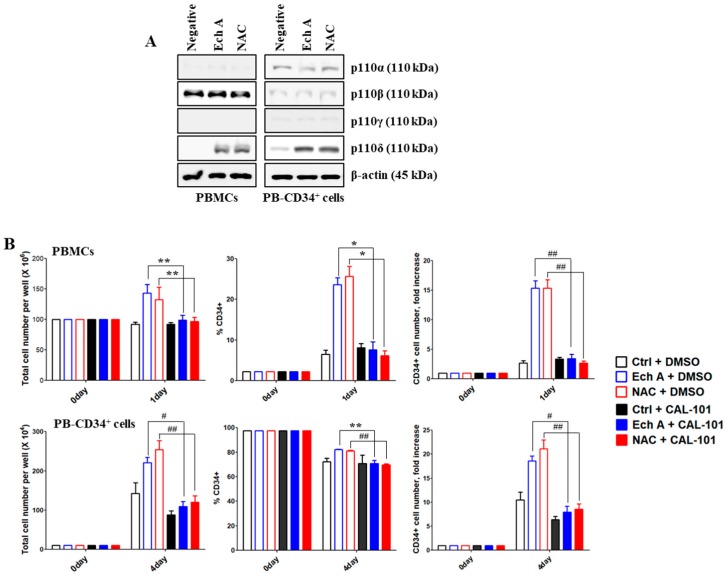
Ech A induces the activation of the PI3K p110δ isoform for PB-CD34^+^ cell expansion. Cells were treated with 10 μM Ech A for 1 day (PBMCs) or 4 days (PB-CD34^+^ cells). For NAC treatment, cells were treated with 5 mM NAC for 4 h, washed, suspended in complete medium, and incubated for an additional 1 day (PBMCs) or 4 days (PB-CD34^+^ cells). (**A**) Total protein was subjected to western blot analysis with the indicated antibodies. (**B**) Cells were pretreated with CAL-101 (20 μM) for 4 h. Total cell number was determined using the ADAM-MC automated mammalian cell counter. For flow cytometric immunophenotypic analysis, cells were stained with CD34-PE, CD38-FITC, CD45-APC, and 7-AAD. Each value was expressed as the mean ± SEM of three independent experiments.

Next, we examined which p110 isoforms were associated with the expansion of PB-CD34^+^ cells, following Ech A or NAC treatment using antibodies, each specific for different p110 isoforms of PI3K. Ech A- or NAC-treated PBMCs or PB-CD34^+^ cells showed a higher expression of p110δ than untreated PB-CD34^+^ cells; however, p110α, p110β, and p110γ expression was not affected by Ech A or NAC treatment (Figure 4A). To identify the role of p110δ in Ech A- or NAC-induced PB-CD34^+^ cell expansion, cells were treated with Ech A or NAC in the presence or absence of the p110δ specific inhibitor, CAL-101. The percentage of CD34^+^ cells and the CD34^+^ cell number in the Ech A or NAC single treatment group (PBMCs, 23.63% ± 1.7%, 15.32 ± 1.27-fold; PB-CD34^+^ cells, 82.07% ± 0.32%, 18.55 ± 1.12-fold) were remarkably decreased by CAL-101 pretreatment (PBMCs, 7.57% ± 1.99%, 3.37 ± 0.74-fold; PB-CD34^+^ cells, 70.67% ± 2.73%, 7.93 ± 1.23-fold) (Figure 4B).

### 2.5. Src/Lyn Is a Major Upstream Signal for Ech A-Induced p110δ-Mediated CD34^+^ Cell Expansion

**Figure 5:**
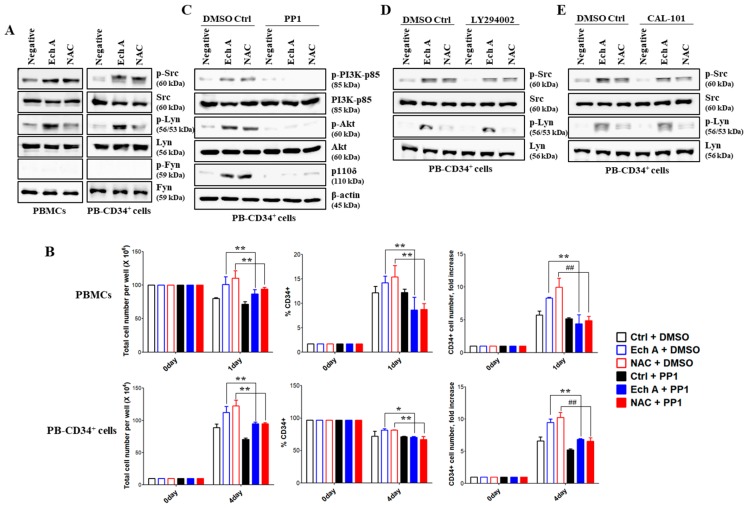
Src/Lyn is major upstream signal for p110δ-mediated CD34^+^ cell expansion by Ech A. Cells were treated with 10 μM Ech A for 1 day (PBMCs) or 4 days (PB-CD34^+^ cells). For NAC treatment, cells were treated with 5 mM NAC for 4 h, washed, suspended in complete medium, and incubated for an additional 1 day (PBMCs) or 4 days (PB-CD34^+^ cells). (**A**,**C**–**E**) Total cell lysates for each condition were harvested and immunoblotted with the indicated antibodies. (**B**–**E**) Cells were pretreated with PP1 (10 μM), LY294002 (10 μM), or CAL-101 (20 μM) for 4 h. (**B**) Total cell number was determined using the ADAM-MC automated mammalian cell counter. For flow cytometric immunophenotypic analysis, cells were stained with CD34-PE, CD38-FITC, CD45-APC, and 7-AAD. Each value was expressed as the mean ± SEM of three independent experiments.

We also investigated Src family kinases associated with the increased expression of p110δ upon Ech A or NAC treatment in PB-CD34^+^ cells. Compared with untreated controls, treatment with Ech A or NAC increased Src phosphorylation levels, but produced little effect on Fyn phosphorylation. Interestingly, Lyn was significantly phosphorylated only by Ech A treatment, but not by NAC (Figure 5A). To identify the requirement for the Src/Lyn pathway in Ech A- or NAC-induced p110δ expression, we used the Src/Lyn inhibitor PP1. The percentages of CD34^+^ cells and the CD34+ cell number in the Ech A or NAC single treatment group (PBMCs, 14.2% ± 1.39%, 8.37 ± 0.13-fold; PB-CD34^+^ cells, 81.57% ± 1.88%, 9.43 ± 0.55-fold) were significantly lower than that in the PP1 pretreatment group (PBMCs, 8.63% ± 2.65%, 4.41 ± 1.40-fold; PB-CD34^+^ cells, 70.27% ± 1.54%, 6.84 ± 0.11-fold) (Figure 5B). To establish a possible link between Ech A- and NAC-induced Src/Lyn phosphorylation and PI3K activation during the ex vivo expansion of CD34^+^ cells, we treated cells with PP1, LY294002, or CAL-101. First, we confirmed the activity of various inhibitors used in this study on each target molecule (Figure S3). As shown in Figure 5C, PP1 dramatically suppressed Ech A or NAC-mediated expression of p110δ and PI3K/Akt activation. On the other hand, LY294002 and CAL-101 did not inhibit Ech A- or NAC-induced Src/Lyn activation (Figure 5D,E). These results suggest that Lyn is activated prior to p110δ and PI3K/Akt and plays a critical role as an upstream regulatory molecule in Ech A-induced ex vivo expansion of PB-CD34^+^ cells.

### 2.6. Ex Vivo Expanded CD34^+^ Cells Maintain Their Colony-Forming Capacity

**Figure 6:**
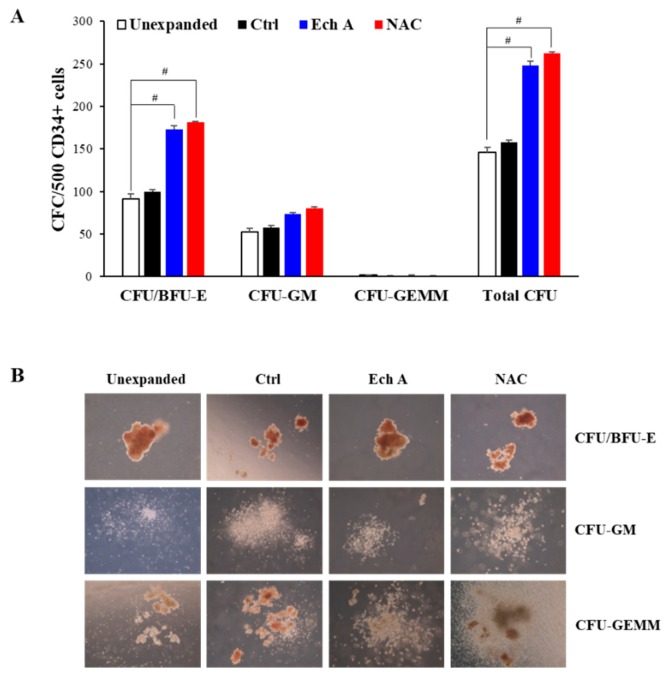
Ex vivo expanded CD34+ cells maintain their colony-forming capabilities. Purified PB-CD34^+^ non-expanded cells, or cells expanded ex vivo in the presence or absence of NAC or Ech A for 4 days were evaluated for pluripotency using hematopoietic colony-forming assays in semi-solid methylcellulose media. (**A**) Colony-forming unit/burst-forming unit-erythroid (CFU/BFU-E), CFU-granulocyte/macrophage (CFU-GM), and CFU-granulocyte/erythroid/macrophage/megakaryocytes (CFU-GEMM) per 500 CD34^+^ cells plated were enumerated after 14 days of culture. (**B**) Representative images of CFU/BFU-E, CFU-GM, and CFU-GEMM for each experimental condition. Magnification: ×200.

As CFU indicates the presence of HSPCs, the colony forming potential (colony-forming unit/burst-forming unit-erythroid (CFU/BFU-E), CFU-granulocyte/macrophage (CFU-GM), and CFU-granulocyte/erythroid/macrophage/megakaryocytes (CFU-GEMM), and total CFU) of freshly isolated CD34^+^ cells or CD34^+^ cells, expanded in the absence or presence of Ech A (10 μM) or NAC (5 mM), was investigated (Figure 6). The frequency of CFU/BFU-E (Ech A, 173.3 ± 3.51; NAC, 181.7 ± 0.58) and total CFU (Ech A, 248.0 ± 5; NAC, 262.7 ± 1.53) in the 10 μM Ech A or 5 mM NAC group was significantly higher than that in the unexpanded (CFU/BFU-E, 91.3 ± 6.11; total CFU, 145.7 ± 6.03) or control expanded group (CFU/BFU-E, 99.3 ± 3.06; total CFU, 157.7 ± 3.06). The frequency of CFU-GM (Ech A, 73.33 ± 2.08; NAC, 80 ± 2) and CFU-GEMM (Ech A, 1.33 ± 0.58; NAC, 1 ± 0) was not vastly different from the unexpanded (CFU-GM, 52.67 ± 4.04; CFU-GEMM, 1.67 ± 0.58) or control group (CFU-GM, 57.3 ± 3.06; CFU-GEMM, 1 ± 0) (Figure 6A). Although the number of CFU/BFU-E was higher in the Ech A and NAC expanded groups, the size of CFU/BFU-E was larger in the unexpanded group (Figure 6B). Therefore, the addition of 10 μM Ech A certainly improved ex vivo expansion of HSPC numbers, but the potency may have slightly decreased.

## 3. Discussion

HSPCs with self-renewal and multipotent capacity offer a valuable source for cell-based therapy and regenerative medicine [36]. Many limitations facing during HSPC transplantations would have been overcome if ex vivo HSPC expansion and maintenance become possible. However, the characteristics of the HSPCs are often altered once they leave the hypoxic bone marrow niche, affecting the quality and quantity of the cultured HSPCs. Studies have shown that the fate of HSPCs is regulated by the microenvironment of the so-called “stem cell niches” that contain oxygen saturations of approximately 5% [7]. The increased oxygen tension in normoxic cultures causes the stem cells to lose their stemness [37]. Various attempts have been carried out to maintain the stemness of HSPCs and to overcome the limitations associated with ex vivo culturing. These include the utility of transcription factors, co-culturing with feeder cells [38], the addition of cytokine cocktails [39], and genetic modification [40]. However, these approaches have some drawbacks, especially when used in a clinical setting. A few previous studies have suggested that the use of antioxidants can ameliorate oxidative stress-mediated damage in cultured HSPCs [41]. In this study, we investigated whether PB-CD34^+^ cells can be efficiently expanded ex vivo by utilizing Ech A, which has been demonstrated as an antioxidant in previous studies [32,34].

HSPCs remain quiescent in the osteoblastic niche—the lowest end of the oxygen gradient in the bone marrow. However, in the oxygen-rich vascular niche, stem cells can proliferate and differentiate closer to blood circulation, resulting in increased intracellular ROS levels. However, an extremely low or high level of ROS would cause impaired repopulation capacity or trigger exhaustion of HSPCs [9]. Physiologically, with the aid of intracellular antioxidant enzyme systems and endogenous antioxidants, HSPCs are able to cope with the damage caused by cumulative ROS [42]. However, the damage is overwhelmed when HSPCs are subjected to ex vivo expansion, wherein a sharp increase in ROS levels is often experienced. It is, therefore, critical to regulate the intracellular ROS levels during ex vivo culturing for the better expansion and maintenance of HSPCs. It has been previously demonstrated that a reduction in intracellular ROS levels by supplementing antioxidants or lowering oxygen tension in cell cultures could improve HSPC expansion, and their engraftment and hematopoietic reconstitution abilities in non-obese diabetic/severe combined immunodeficiency (NOD/SCID) mice [43]. Consistent with this finding, PB-CD34^+^ cells provoked aberrant ROS generation in the normoxic culture (Figure 1A). PB-CD34^+^ cells exhibited higher ROS levels than BM-CD34^+^ cells (Figure 1A). ROS is a critical mediator of HSPC quiescence with p38-MAPK. Higher ROS levels can activate the p38-MAPK pathway, which in turn can promote phosphorylation of p38-MAPK [44]. Our results also showed that PB-CD34^+^ cells can upregulate phospho-p38-MAPK and phospho-JNK, likely due to high ROS levels (Figure 2A). p38-MAPK plays a role in hematopoiesis regulation, particularly in erythropoiesis and granule formation [45]. Recently, p38-MAPK was identified as an intrinsic modulator that can negatively regulate HSPC self-renewal [46]. Therefore, both ROS and p38-MAPK have been shown to play an important role in maintaining HSPC quiescence. Several studies have indicated that HSPCs lose their ability to regenerate due to elevated ROS levels and specific phosphorylation of p38-MAPK. Pharmacological inhibition of ROS or p38-MAPK activity can restore HSPC function in the ROS^high^ mouse population and rescue these mice from bone marrow damage [7]. Treatment of Ech A and NAC potently suppressed the activation of p38-MAPK and JNK, and the p38-MAPK inhibitor SB203580 and the JNK inhibitor SP600125 promoted CD34^+^ cell expansion, potentially by inhibiting differentiation (Figure 2). It is noteworthy that the suppression of ROS by Ech A ameliorated the activation of p38-MAPK and JNK, suggesting that Ech A acts as an efficient antioxidant in the ex vivo culture of PB-CD34^+^ cells, and subsequently promotes CD34^+^ cell expansion.

SFKs in hematopoietic tissues can function as primary regulatory factors, as described in the first *p60-Src* gene perturbation experiment to confirm its role in osteopetrosis development [47]. Subsequent studies revealed SFK activities in B cells, bone marrow, obese cell lines, and Lyn-expressing HSPCs in all blood cell lines except T cells [19]. In some studies, Lyn was shown to play negative roles in monocyte production and plasma cell function, as revealed in *Lyn*^-/-^ mice by M-phi tumorigenesis [48] and IgM hyperglobulinemia [20]. Although not widely studied, SFKs have also been suggested as important regulators of erythropoiesis. Avian Src was originally discovered as an oncogene that promotes sarcoma and erythroleukemia [49]. Interestingly, *Lyn*^–/–^ mice among the SFK gene-deficient mice showed an age-dependent increase in the production of splenic erythroblasts [50]. In BM-derived cultures, early-stage *Lyn*^–/–^ erythroblasts exhibited a reduced ability to expand and develop beyond the Kit^+^CD71^+^ stage [51]. Therefore, SFKs, such as Lyn and Src, are major signaling mediators that modulate diverse stimuli to regulate differentiation, migration, proliferation, apoptosis, and metabolism. However, there is limited information regarding the precise role Lyn/Src and their underlying molecular mechanisms in the ex vivo expansion of PB-CD34^+^ cells. PI3K/Akt signaling can be activated by the downregulation of PTEN by BCR–ABL2. PTEN is a lipid phosphatase that interferes with PI3K signaling by dephosphorylating phosphatidylinositol-3,4,5-trisphosphate. Class I PI3Ks consist of four different catalytic isoforms (p110α, p110β, p110γ, and p110δ) and two standard regulatory subunits (p85 and p101) [52]. PI3K plays an important role in HSPC maintenance and regulation of lineage development [53]. In this study, we found, for the first time to our knowledge, that Ech A activates Lyn, which in turn upregulates p110δ expression, and suppresses ROS production and p38-MAPK/JNK phosphorylation, resulting in enhanced ex vivo expansion of PB-CD34^+^ cells (Figure 4 and Figure 5). Furthermore, the addition of Ech A increased CFU/BFU-E producing cell numbers (Figure 6), which suggests that an appropriate use of Ech A is advantageous for CD34^+^ cells to maintain self-renewal potential during ex vivo expansion.

**Figure 7:**
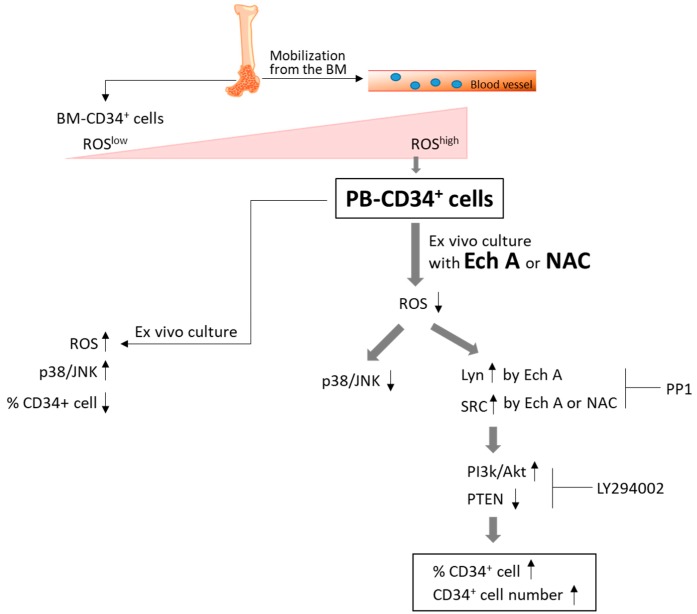
Schematic representation of Ech A and NAC in the ex vivo expansion of PB-CD34^+^ cells. Mobilized PB-CD34^+^ cells showed higher ROS levels than BM-CD34^+^ cells. Ech A suppressed intracellular ROS production in PB-CD34^+^ cells. Ech A also increased Lyn/Src phosphorylation and PI3K/Akt activation. Our results suggest that Ech A promotes ex vivo expansion of CD34^+^ cells through Lyn/Src-mediated p110δ expression.

Our findings, as summarized in Figure 7, demonstrate that Ech A can effectively inhibit ROS production in PB-CD34^+^ cells. ROS-mediated p38-MAPK/JNK activation can reduce the number of CD34^+^ cells, and decrease the self-renewal of PB-CD34^+^ cells, which was reversed by Ech A treatment. Our results also demonstrate a novel Lyn-mediated p110δ expression by Ech A in PB-CD34^+^ cells, although the precise molecular mechanism of Ech A-induced ex vivo expansion, especially how Lyn and p110δ are coordinated in PB-CD34^+^ cells in response to Ech A, remains unclear. Taken together, Ech A was found to be an effective agent for promoting cell proliferation and maintaining the stemness of HSPCs. Ech A is beneficial for CD34^+^ cells to maintain their self-renewal potential and function during the ex vivo expansion, and possibly during in vivo expansion of HSPCs.

## 4. Materials and Methods

### 4.1. Isolation of PBMCs and Purification of PB-CD34^+^ Cells

This study was conducted using samples from healthy donors and patients, and the study protocol was approved by the Institutional Review Board at the Kosin University College of Medicine. Informed consent for the study was obtained from all donors. Purified BM- or PB-CD34^+^ cells were obtained either from BM (Lonza, Basel, Swizerland) or from small aliquots of mobilized PB from healthy donors and patients. CD34^+^ cells from PBMCs were immunoselected using the MACS CD34 MicroBead kit UltraPure (Miltenyi Biotec, Bergisch Gladbach, Germany). Human CD34^+^ cells were enriched from PBMCs by magnetic bead positive selection using Miltenyi immunomagnetically activated cell sorter (MACS; Miltenyi Biotec). PB-CD34^+^ cells were then stained for CD45, and the CD34^+^ purity of greater than 95% was reanalyzed by flow cytometry. PB-CD34^+^ cells were expanded in serum-free medium (SFEM) (Stem Cell Technologies, Vancouver, Canada) supplemented with 50 ng/mL rhSCF (PeproTech, Rocky Hill, NJ, USA), 10 ng/mL rhIL3 (PeproTech), and 25 μg/mL LDL (Stem Cell Technologies).

### 4.2. Chemicals and Reagents

Ech A was received as a gift from G.B. Elyakov, Pacific Institute of Bioorganic Chemistry, Vladivostok, Russia. PPI, SP600125, SB203580, LY294002, and CAL-101 were purchased from Selleck Chemicals (Houston, TX, USA). NAC was obtained from Sigma-Aldrich (St. Louis, MO, USA).

### 4.3. Immunophenotypic Analysis by Flow Cytometry

PBMCs, PB-CD34^+^ cells, and BM-CD34^+^ cells were tested for cell surface antigen expression by immunofluorescence and flow cytometric analysis. Cells were harvested and rinsed with PBS, following which CD34-PE (Miltenyi Biotec, #130-113-179, 1:50), CD38-FITC (Miltenyi Biotec, #130-113-426, 1:50), CD45RA-APC (Miltenyi Biotec, #130-117-742, 1:50), and 7-AAD (eBioscience, Waltham, MA, USA, 3 μL/10^5^ cells) were added. Thereafter, cells were incubated at 4 °C for 10 min in the dark. Finally, the stained cells were analyzed using a BD Accuri^TM^ C6 (BD Biosciences).

### 4.4. Determination of Intracellular ROS Production

The intracellular accumulation of ROS was determined by flow cytometry after staining with the fluorescent probe DCFH-DA (10 μM, 2′,7′-dichlorodihydro-fluorescein diacetate; Molecular Probes, Invitrogen, Milan, Italy). DCFH-DA was deacetylated in cells by esterase to a nonfluorescent compound DCFH, which remains trapped within the cell and is cleaved and oxidized by ROS in the presence of endogenous peroxidase to a highly fluorescent compound DCF (2′,7′-dichlorofluorescein). Cells were incubated with 10 μM DCFH-DA for 30 min at 37 °C, washed, and resuspended in PBS. ROS levels were monitored using a BD Accuri^TM^ C6 (BD Biosciences).

### 4.5. Immunoblotting

Cells were lysed in RIPA (radioimmunoprecipitation assay) buffer (Elpis Biotech, Daejeon, Korea) supplemented with a protease inhibitor cocktail (Calbiochem, La Jolla, CA, USA) and protein phosphatase inhibitors (Calbiochem). Protein concentrations were determined using a BCA assay kit (Pierce, Rockford, IL, USA). Proteins (10 μg/sample) were resolved in an SDS-PAGE gel and transferred to a nitrocellulose membrane (Millipore Corp., Billerica, MA, USA). Membranes were blocked with 5% skim milk prior to western blot analysis. Chemiluminescence was detected using an ECL kit (Advansta Corp., Menlo Park, CA, USA) and the Amersham Imager 600 (GE Healthcare Life Sciences, Little Chalfont, UK). The following primary antibodies were used on fresh individual membranes to minimize interference by stripping and reprobing: phospho-JNK (Thr^183^/Tyr^185^), JNK, phospho-p38 MAPK (Thr^180^/Tyr^182^), p38 MAPK, phospho-ERK1/2 (Thr^202^/Tyr^204^), ERK1/2, phospho-Src (Tyr^416^), Src, phospho-Lyn (Tyr^507^), Lyn, phospho-Akt (Ser^473^), Akt, phospho-PI3K p85 (Tyr^458^/Tyr^199^), PI3K p85, PI3K p110α, PI3K p110β, PI3K p110γ, PI3K p110δ, phospho-PTEN (Ser^380^/Thr^382/383^), PTEN, and Fyn (Cell Signaling Technology, Beverly, MA, USA); and β-actin and phospho-Fyn (Santa Cruz Biotechnology, Santa Cruz, CA, USA). All the raw data from immunoblotting experiments are presented in Figure S4.

### 4.6. Colony-Forming Cell (CFC) Assay

CD34^+^ cells (500 cells/dish) were plated in methylcellulose medium (Methocult H4100, STEMCELL Technologies, Inc., Canada) supplemented with SCF (50 ng/mL), IL-3 (10 ng/mL), GM-CSF (10 ng/mL) (PeproTech), and EPO (1 U/mL) in 35-mm culture dishes and incubated at 37 °C in a 5% CO_2_ chamber for 14 days. Colonies were counted and analyzed using a scoring grid and an inverted microscope (Olympus, Japan). Colonies were classified based on their morphology as colony-forming unit/burst-forming unit-erythroid (CFU/BFU-E), CFU-granulocyte/macrophage (CFU-GM), and CFU-granulocyte/erythroid/macrophage/megakaryocytes (CFU-GEMM).

### 4.7. Statistical Analysis

The data were analyzed using Student’s *t*-tests and one-way analyses of variance (ANOVA) with GraphPad Prism software, and are presented as standard error of the mean (SEM), unless otherwise stated. Statistical significance was defined as * *p* < 0.01, ** *p* < 0.05, # *p* < 0.001, and ## *p* < 0.005.

## Supplementary Materials

The following are available online at https://www.mdpi.com/1660-3397/17/9/526/s1, Figure S1: Dose-dependent effect of Ech A or NAC on ex vivo expansion of PBMCs; Figure S2: Ech A recovered PB-CD34^+^ cell expansion that was suppressed by H_2_O_2_ treatment; Figure S3: Each inhibitor was confirmed to work as expected; Figure S4: Raw data from immunoblotting experiments.
