# Supplementary material for: Echinochrome A Promotes Ex Vivo Expansion of Peripheral Blood-Derived CD34+ Cells, Potentially through Downregulation of ROS Production and Activation of the Src-Lyn-p110δ Pathway
Source: Mar Drugs. 2019 Sep 9;17(9):526. doi: 10.3390/md17090526 (PMC6780187; doi:10.3390/md17090526)
Supplement: Supplementary file 1 [file marinedrugs-17-00526-s001.pdf]

Fig. S1

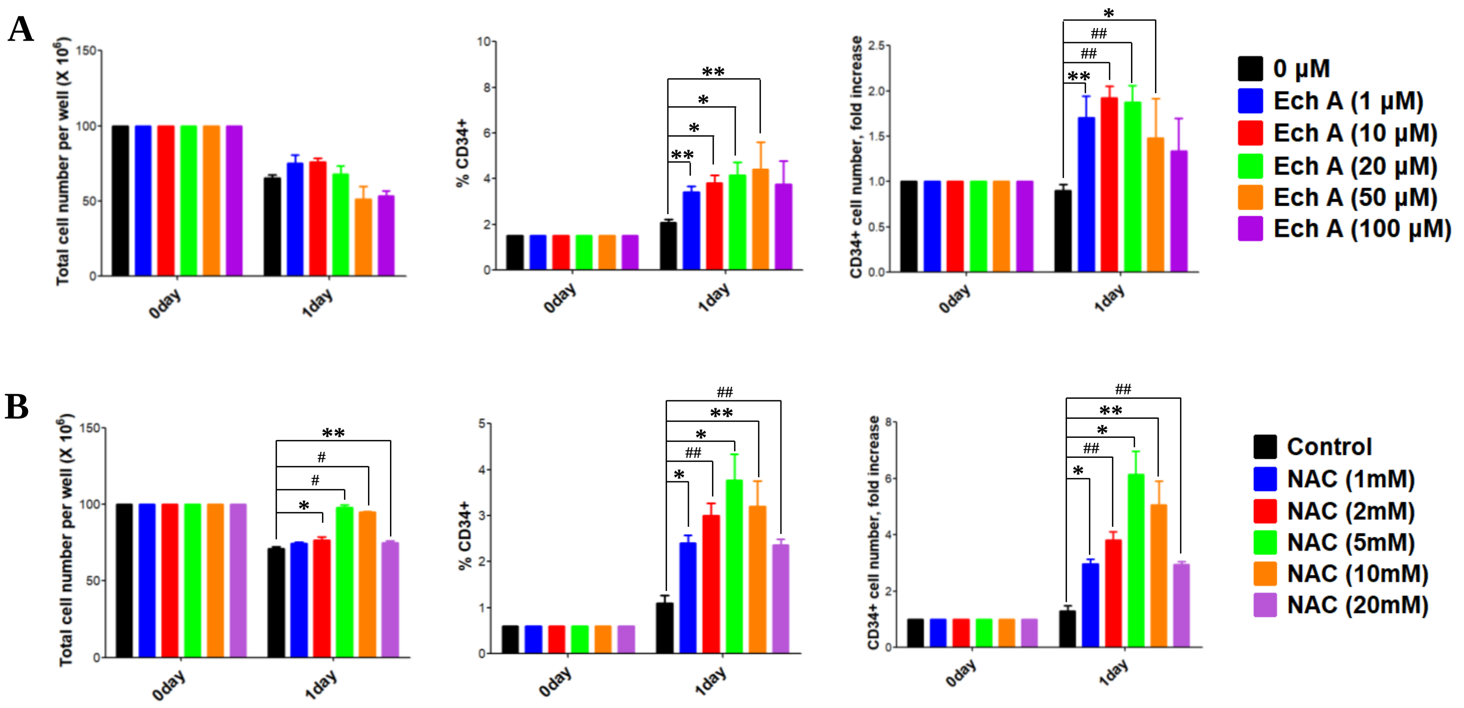

Figure S1. Dose-dependent effect of Ech A or NAC on ex vivo expansion of PBMCs. (A) PBMCs were treated with 1, 10, 20, 50, and 100 μM Ech A for 24 h. (B) PBMCs were treated with 1, 2, 5, 10, and 20 mM NAC for 4 h. After washing, cells were suspended in complete medium and incubated for an additional 20 h. Total cell number was measured using the ADAM-MC automated mammalian cell counter. For flow cytometric immunophenotypic analysis, cells were stained with CD34-PE, CD38-FITC, CD45-APC, and 7-AAD. Each value was expressed as the mean ± SD of three independent experiments.

Fig. S2

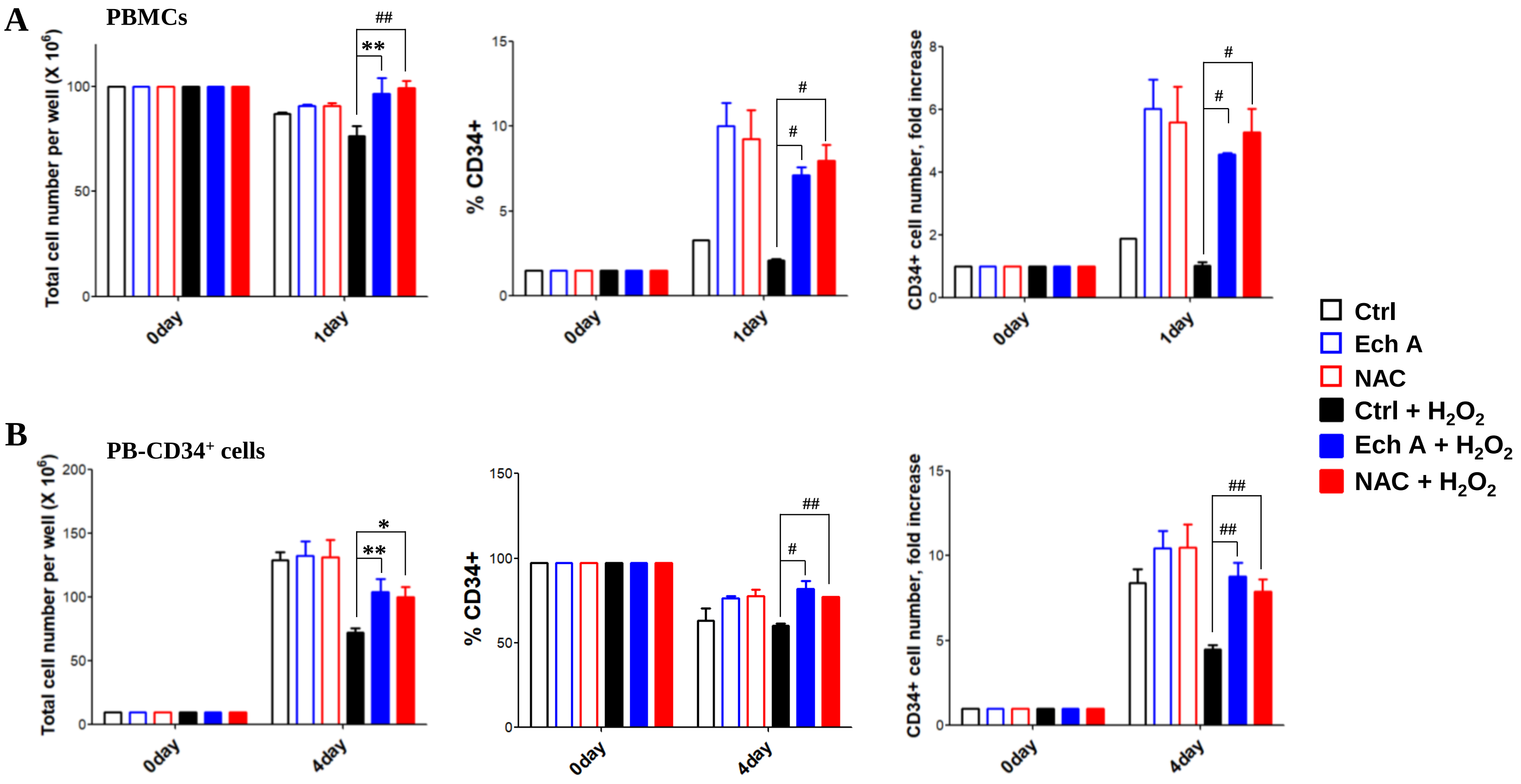

Figure S2. Ech A recovered PB-CD34$^+$ cell expansion that was suppressed by H_2O_2 treatment. Cells were treated with 10 µM Ech A for 1 day (PBMCs; A) or 4 days (PB-CD34$^+$ cells; B). For H_2O_2 treatment, cells were treated with 100 µM H_2O_2 for 2 h, washed, suspended in complete medium, and incubated for an additional 1 day (PBMCs) or 4 days (PB-CD34$^+$ cells). Total cell number was determined using the ADAM-MC automated mammalian cell counter. For flow cytometric immunophenotypic analysis, cells were stained with CD34-PE, CD38-FITC, CD45-APC, and 7-AAD. Each value was expressed as the mean ± SD of three independent experiments.

Fig. S3

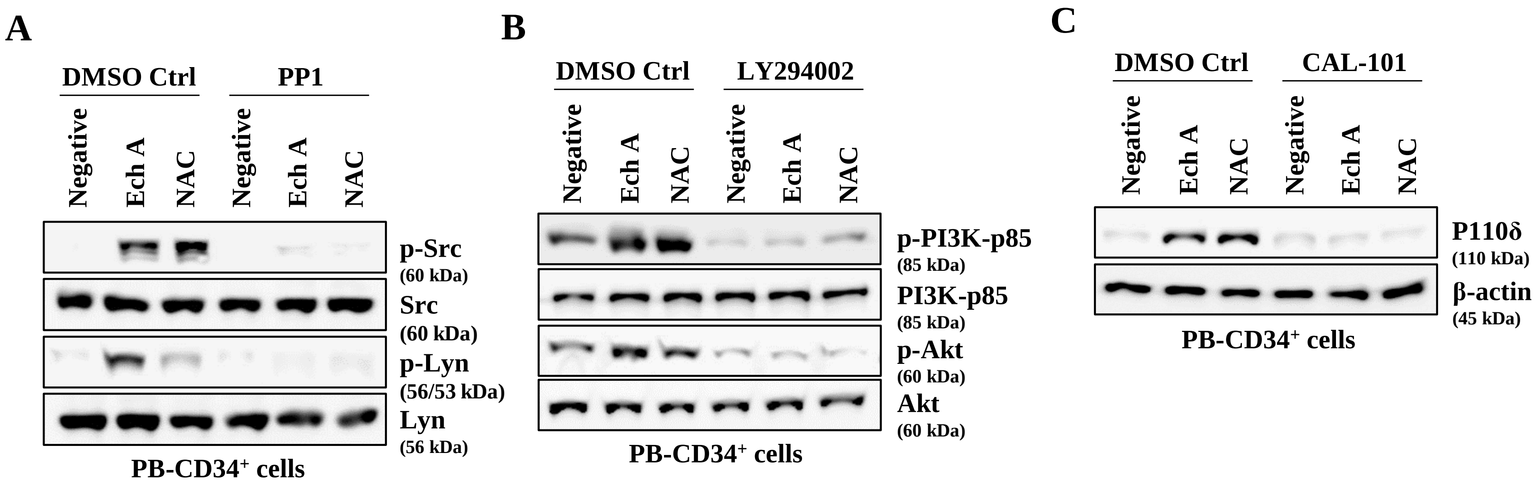

Figure S3. Each inhibitor was confirmed to work as expected. PBMC-derived $CD34^+$ cells (5×10^4/well) were pretreated with PP1 (10 µM; A), LY294002 (10 µM; B), or CAL-101 (20 µM; C), for 4 h. Cells were then washed and treated with Ech A (10 µM) or NAC (5 mM) for 4 days. Total cell lysates for each condition were immunoblotted using the indicated antibodies. β-actin served as an internal control.

Fig. S4

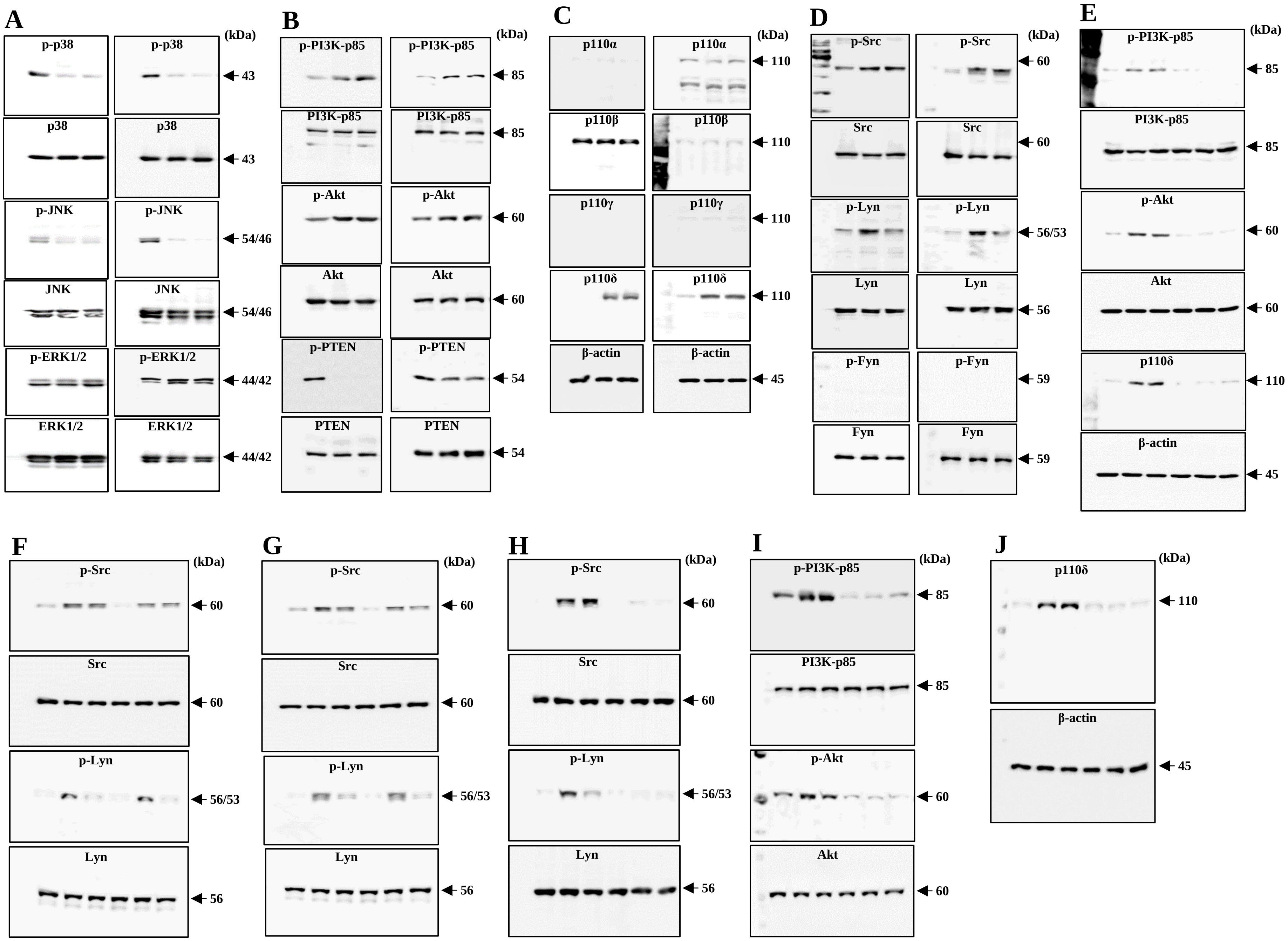

Figure S4. Raw data from immunoblotting experiments. Raw data of Fig. 2A (A), Fig. 3A (B), Fig. 4A (C), Fig. 5A (D), Fig. 5C (E), Fig. 5D (F), Fig. 5E (G), Supple. Fig. 3A (H), Supple. Fig. 3B (I), and Supple. Fig. 3C (J) were presented.
